# Innate Immunity Mechanisms in Marine Multicellular Organisms

**DOI:** 10.3390/md20090549

**Published:** 2022-08-25

**Authors:** Svetlana V. Guryanova, Tatiana V. Ovchinnikova

**Affiliations:** 1 M.M. Shemyakin and Yu.A. Ovchinnikov Institute of Bioorganic Chemistry RAS, 117997 Moscow, Russia; 2 Medical Institute, Peoples’ Friendship University of Russia, 117198 Moscow, Russia; 3 Department of Bioorganic Chemistry, Faculty of Biology, Lomonosov Moscow State University, 119234 Moscow, Russia; 4 Department of Biotechnology, I.M. Sechenov First Moscow State Medical University, 119991 Moscow, Russia

**Keywords:** innate immunity, PRR, TLR, NLR, RLR, CLR, marine invertebrates, mucous, antimicrobial peptides, AMP

## Abstract

The innate immune system provides an adequate response to stress factors and pathogens through pattern recognition receptors (PRRs), located on the surface of cell membranes and in the cytoplasm. Generally, the structures of PRRs are formed by several domains that are evolutionarily conserved, with a fairly high degree of homology in representatives of different species. The orthologs of TLRs, NLRs, RLRs and CLRs are widely represented, not only in marine chordates, but also in invertebrates. Study of the interactions of the most ancient marine multicellular organisms with microorganisms gives us an idea of the evolution of molecular mechanisms of protection against pathogens and reveals new functions of already known proteins in ensuring the body’s homeostasis. The review discusses innate immunity mechanisms of protection of marine invertebrate organisms against infections, using the examples of ancient multicellular hydroids, tunicates, echinoderms, and marine worms in the context of searching for analogies with vertebrate innate immunity. Due to the fact that mucous membranes first arose in marine invertebrates that have existed for several hundred million years, study of their innate immune system is both of fundamental importance in terms of understanding molecular mechanisms of host defense, and of practical application, including the search of new antimicrobial agents for subsequent use in medicine, veterinary and biotechnology.

## 1. Introduction

The first multicellular organisms arose in the world’s oceans, and those that were able to develop mechanisms for maintaining homeostasis and pass them on to subsequent generations have survived to this day. Many of them, such as coelenterates and tunicates, are over five hundred million years old.

Innate immunity reacts immediately to stress signals and pathogens and reflects a significant part of infectious agents in barrier tissues, including mucous membranes. Adaptive immunity, which first appeared in cartilaginous fish, develops more slowly, includes the formation of immunoglobulins and specialized memory cells, and is aimed at destroying pathogens passed through barrier tissues.

The existence of primitive metazoans over several hundred million years proves the effectiveness of innate immunity against the variability of members of the microbiological community. The innate immune system provides an adequate response to pathogens and tolerance to harmless microflora through pathogenic pattern receptors (PRRs), located on the surface of cell membranes and within cells that recognize pathogen-associated molecular patterns (PAMPs). Another important function of PRRs is the maintenance of homeostasis in stressful situations and the utilization of dead cells through the recognition of damage-associated molecular patterns (DAMPs) of endogenous origin [1]. The main PRR classes are as follows: Toll-like receptors (TLRs), Nod-like receptors (NLRs), retinoic acid-induced gene I (RIG-I)-like receptors (RLRs), C-type lectin receptors (CLRs), and some others [2]. The structures of PRRs are formed by several evolutionarily conserved domains, with a fairly high degree of homology in representatives of different species [2].

## 2. Toll-Like Receptors

**Figure 1:**
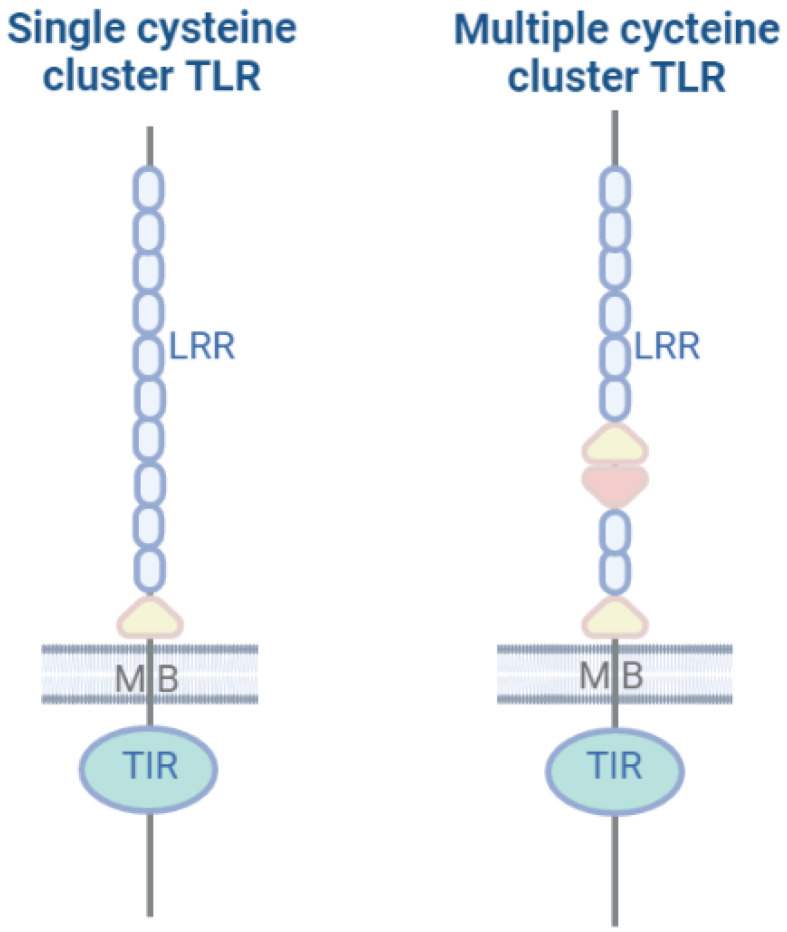
Structures of TLRs. Leucine-rich repeat extracellular domain (LRR), membrane spanning transmembrane domain (MB), and cytoplasmic domain (TIR). LRR domains containing cysteine residues in the C-terminal part (LRRCT) are depicted as yellow triangles. LRR domains containing cysteine residues in the N-terminal part (LRRNT) are depicted as pink triangles. V-type TLRs have only one LRRCT (single cysteine cluster) located next to the TIR domain; P-type TLRs have more than one LRRCT and sometimes an LRRNT (multiple cysteine cluster) domain.

Toll-like receptors (TLRs) are the most ancient class of PRRs, appearing more than 650 million years ago [3]. The TLR structure is represented by homo- and heterodimers that recognize bacterial and viral pathogens, as well as endogenous danger signals. In humans, TLRs are represented by 10 variants, consisting of 700–1100 amino acid residues. TLRs more often function as homodimers, while heterodimers also exist, and the recruitment of other proteins expand the ability of TLRs to recognize ligands. For example, the human TLR4 uses the MD2 and CD14 proteins to recognize LPS, with the LPS structure being critical [4,5]. TLRs consist of an extracellular leucine-rich repeat (LRR) domain for DAMP/PAMP recognition, a transmembrane domain, and a cytoplasmic Toll/IL-1 signal transduction receptor (TIR) domain. Some LRR domains contain cysteine residues in the N-terminal part (LRRNT) or an additional C-terminal (LRRCT) residue, in which case a multiple cysteine cluster is formed. Another nomenclature is also used, which is as follows: (1) the V-type for the single cysteine cluster has only one LRRCT located near to the TIR domain; (2) the P type for the multiple cysteine cluster has more than one LRRCT, and sometimes the LRRNT domain. The proteins that lack the LRR or TIR domain are not classified as TLR receptors and are considered as TLR-like proteins (Figure 1).

LRR domains are also present in other innate immune receptors that belong to the NLR family, as well as in a wide range of transmembrane proteins involved in intercellular contacts during development [6,7].

The LRR domain of TLR recognizes PAMP or DAMP and converts the received signal via the cytoplasmic TIR domain into activation of intracellular pathways. TIR domains recruit signal adapters MyD88, TIRAP, TRAM and/or TRIF, then various kinases (IRAK4, IRAK1, IRAK2, TBK1 and IKK1) and ubiquitin ligases (TRAF6 and pellino 1). This chain of protein–protein interactions creates a signal transduction pathway that links the activated receptor to its response. The final targets of TLRs are DNA-binding transcription factors (such as NF-kB, IRFs, etc.), which activate specific gene expression patterns in the nucleus, resulting in the production of antimicrobial peptides (AMPs), pro-inflammatory cytokines and chemokines, including tumor necrosis factor-α (TNF-α), interleukins (IL) IL-1β, IL6 and others [8,9,10]. TLRs are located on the outer membrane and on endosome membranes. Only surface TLR1, TLR2, and TLR4 could induce ROS production in macrophages, while stimulation of endosomal TLRs (TLR3/7/8/9) did not induce ROS [11]. Thus, stimulation of surface TLRs increases microbicidal activity.

The first TLRs were identified in *Drosophila* [12,13,14]. In *Drosophila*, TLR1 controls the immune response to Gram-positive bacteria and fungi by distinguishing peptidoglycans and activating a signaling pathway that has been conserved throughout evolution [15]. The *Drosophila* genome encodes eight additional Toll-related receptors, most of which are involved in the development process [16]. Using the amino acid sequence of *Drosophila* TLR1, related sequences were found in the Human Genome Project database and Toll-like receptors were identified [17,18].

The diversity of TLRs varies greatly among animal species; the ligands defined by these TLRs also differ. Recently, phylogenetic studies of the TLR gene family have revealed that among metazoans, TLRs have not been found outside of *Cnidaria* and *Bilateria* [19].

In marine fish *Gadiformes morhua*, 42 homologues of the human TLRs were found, and an increase in temperature from 2 °C to 6 °C contributed to a significant increase in the TLR5 gene expression and a decrease in TLR21 expression [20]. In the genome of echinoderms, the purple sea urchin *Strongylocentrotus purpuratu* has 222 Toll-like receptor (TLR) genes and a corresponding increase in directly related signaling adapters [21]. The purple sea urchin *S. purpuratu* is found in coastal areas of the Pacific Ocean along the west coast of the United States and Canada, and has a lifespan similar to that of a human, ranging from 50 to 100 years [22]. Sea urchins belong to the phylum Echinodermata, and to the group of deuterostomes, which also includes the phylum Chordata.

In phylogenetically distant animals, TLRs perform different functions to achieve the same goal of protecting against pathogens during infection (Table 1).

TLRs are activated not only when pathogens are recognized. Cell damage or destruction (DAMP) signals, when intracellular components are released into the environment, specifically activate TLR [23], while extracellular matrix degradation products can also act as damage signals [24]. The significance of these signals may lie in the involvement of professional phagocytes for the elimination of destroyed cells and subsequent tissue regeneration. It should be noted that excessive activation of TLR4 in mammals contributes to tissue scarring, with loss of specific functions [25].

## 3. Nod-Like Receptors

**Figure 2:**
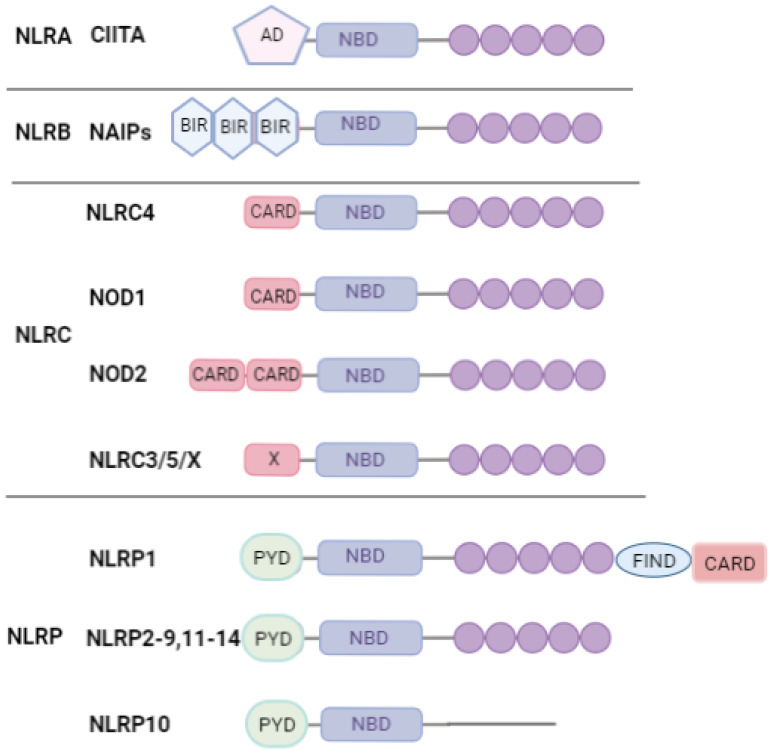
NLR family. The NLR family is subdivided into the following four subgroups: NLRA, NLRB, NLRC, and NLRP, depending on the nature of the N-terminal domain, consisting of the transactivation domain (AD), the baculovirus IAP repeat (BIR), the caspase activation and recruitment domain (CARD), and the pyrine domain, respectively. (PYD). The FIND domain is also present in NLRP1. PYD and CARD are death domains (DD) that appear to mediate homotypic domain interactions. LRR domains represented as circles.

Nod-like receptors (NLRs) are intracellular PAMP and DAMP recognition receptors and are multidomain proteins. The human NLR family is represented by twenty-two proteins, in which the following three parts can be distinguished: (1) the C-terminal agonist-receptive/ligand-binding leucine rich repeat (LRR) domain; (2) the central nucleotide-binding and responsible for the oligomerization domain NOD (NBD/NACHT); (3) N-terminal signaling caspase activation and recruitment domain (CARD) (Figure 2) [26,27].

**Figure 3:**
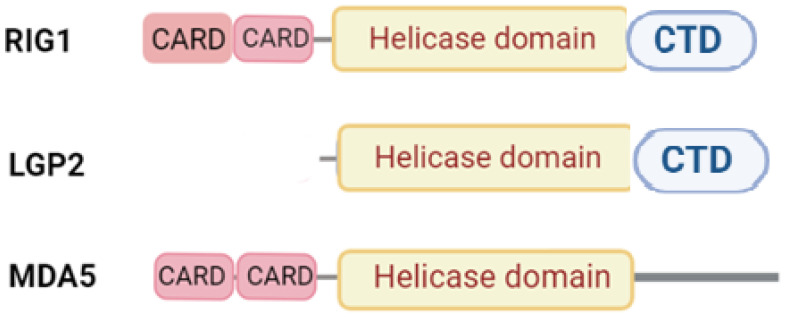
RIG-I-like receptor (retinoic acid-inducible gene-I-like receptors, RLRs) family: retinoic-acid inducible gene 1 (RIG-I), laboratory of genetics and physiology 2 (LGP2); melanoma differentiation-associated 5 (MDA5). All RLRs have a central helicase domain. C-terminal domain (CTD) and the caspase activation and recruitment domain (CARD) may be also present.

Among the NLRs, NLRP1, NLRP3, NLRP6, NLRP7, NLRP12, NLRC4, and NAIP have been reported to operate via inflammasomes (Figure 3). Other NLRs, such as NOD1, NOD2, NLRP10, NLRX1, NLRC5, and CIITA, do not interact directly with inflammatory caspases, but instead activate nuclear factor-κB (NF-κB), mitogen-activated protein kinases (MAPKs) and interferon regulatory factors (IRF) that contribute to the stimulation of innate immunity [28].

Among all the PRRs, NLRs represent the largest and most diverse family, both structurally and functionally, as well as in regard to the signal repertoire that they recognize [29].

NLRs were first described in plants as pathogen resistance factors, and the genes encoding them were named R-genes [30]. Later, their analogues were found in humans. A genome-wide study of 38 representative model organisms, including major taxa (eubacteria, archaebacteria, protists, fungi, plants, and metazoans), showed that two major domains, NBD and LRR, existed prior to the separation of prokaryotes and eukaryotes [31]. The authors of the study conclude that the similarity of the innate immune systems of plants and animals was formed as a result of convergent evolution of their independent origins [31]. The independent origin of NLRs in different animal species explains the absence of NLRs in fruit flies, and the presence of 3 of them in sea anemones (*Nematostella vectensis*), and a significant number of 203 in sea urchins [32,33,34]. In *Drosophila*, the function of the immune response to pathogens is performed by TLRs, carrying out the Toll-mediated NF-κB response [34]. In sea urchins, NLRs are located primarily in the gut, and the diversity of TLRs and NLRs may be in response to the diversity of microorganisms in the habitat [32].

Whole genome sequencing of the sponge *Amphimedon queenslandica* revealed the presence of a large set of genes that contained the NACHT domain and 135 NLR domains. Approximately half of them have a tripartite architecture that includes the N-terminal CARD or DEATH domain [35]. In the marine coelenterate *Hydractinia symbiolongicarpus*, transcriptome analysis revealed both canonical and non-canonical NOD-like receptors, while neither canonical Toll-like receptors (TLRs) nor any transmembrane proteins with a Toll/interleukin-1 (TIR) domain have been identified [36].

Recently, genome-wide studies of marine multicellular organisms provided new data on innate immune receptors, but the interpretation of the obtained results may vary based on different definitions of NLRs. Thus, in the study of the genome of *Hydra magnipapillata*, 290 NLR-like genes were reported (Table 2) [37]. However, if one adheres to the universal nomenclature and NLR definition [26] adopted by the HUGO Gene Nomenclature Committee, NLR is designated as a gene that contains a “nucleotide-binding domain and a leucine-rich repeat”. This highlights the definition of two evolutionarily conserved domains, reflecting the non-homologous similarity of animal NLRs to plant NLRs [26,35]. Thus, the discovered hydra genes that contain NACHT, NB-ARC, CARD, and DD domains cannot be considered as full-fledged NLRs, due to the absence of the LRR domain. However, the 290 structures found that lack LRR domains and are located in the hydra ectoplasm protect hydra against microorganisms. The host defense is not based on the detection of pathogenicity patterns using the LRR domain, but relies on the response of the NACHT, NB-ARC, CARD and DD domains to stress molecules arising from pathogen invasion.

The data obtained are confirmed by recent studies showing that members of the NLR family NOD1 and NOD2 are activated upon interaction with the endogenous metabolite sphingosine-1-phosphate (S1P) [38]. S1P binds to NBD and activates RIP2-mediated signaling, which differs from peptidoglycan sensing via NOD1/2 LRR domains and points to a different mechanism for NOD1/2 activation by S1P. A second messenger, S1P, has pleiotropic effects both extracellularly and intracellularly, regulating various processes, including immune cell trafficking, inflammation, and apoptosis [39]. Taking into account the fact that S1P is structurally and metabolically conserved throughout evolution [40], Pie et al. concluded that “cytosolic S1P generated when cellular homeostasis is disturbed represents an endogenous stress-associated molecular pattern (SAMP)” [38]. S1P is generated in the cytosol upon induction of cellular stress without significant cell death, in contrast to the canonical molecular patterns associated with damage or danger (DAMP) released after cell lysis [41].

Thus, based on recent achievements, it can be argued that the originally identified functions of NLRs to determine pathogenicity patterns inherent in microorganisms are not the only functions. Intracellular recognition of danger signals via second messengers, such as S1P binding to the NBD domain, is another function of NLR and perhaps a more ancient one. The mechanism that allows a cell to detect the presence of any pathogen by its metabolic products or by secondary messengers that occur in the cell during invasion, regardless of PAMP, can be considered universal, protecting the body from microorganisms whose PAMP cannot be recognized. It becomes clear why under the influence of NLRs inducers, for example, of MDP or GMDP, anti-infective protection against a wide class of pathogens of bacterial or viral etiology increases [42,43,44,45,46,47]. Given the diverse functions of NLRs in maintaining homeostasis, NLRs are considered to be ancient guardians of the innate immune system [48].

## 4. RIG-I-Like Receptors

RIG-I-like receptors (retinoic acid-inducible gene-I-like receptors, RLRs) are cytosolic sensors of RNA-containing viruses and are represented by the following three proteins: RIG-I (retinoic-acid inducible gene), MDA5 (melanoma differentiation-associated 5) and LGP2 (laboratory of genetics and physiology 2) [49].

A common feature of all the three RLRs is the presence of a central helicase domain with the ATPase activity that unwinds RNA. The C-terminal domain (CTD) also binds viral RNA (Figure 3).

The different CTDs of the three RLRs determine the type of RNA they can bind to. For example, RIG-I binds short <2000 bp. single- or double-stranded RNAs, MDA5 binds preferentially double-stranded RNAs >2000 bp; LGP2 binds to double-stranded RNA with blunt ends of different lengths [50,51,52]. RIG-I and MDA5 also have two N-terminal CARDs (caspase active recruitment domains) that are required to initiate downstream signaling. LGP2 lacks CARD signaling domains, allowing it to be downregulated by RIG-I [49]. Binding of RLRs to a ligand initiates signaling cascades, resulting in type 1 interferons (IFNs) [49]. Type I IFNs are important cytokines in the antiviral system of innate immunity [53]; their synthesis is regulated by the following two signaling cascades: (1) a signal induced by pathogens with IFN production; (2) a signal mediated by the IFN receptor [54]. Viral RNA binding by RLRs activates transcription factors, such as the activating transcription factor (ATF)⁄C-JUN, the nuclear factor κB (NF-κB), and the IFN regulatory factor (IRF). These factors activate the IFN-b transcription through interaction with the IFN-b RNA polymerase promoter region and promote the transcription of hundreds of genes [55]. RLR recognition of foreign RNA and subsequent signaling, resulting in virus inactivation, is an important defense mechanism against viral infections. Activated RIG-I and MDA5 signaling pathways are known to interact with mitochondrial antiviral signaling proteins (MAVS), and this interaction induces the recruitment of downstream signaling molecules, with MAVS being a key adapter for RLR signaling [56,57].

In invertebrates, antiviral protection is realized mainly through RNA interference [58], but it turned out that the RLR signaling pathway also plays an indispensable role in the host antiviral immunity [59,60]. At the same time, the signal activation pathway in the mollusk was similar to the RLR activation pathway in humans. It was found that, similar to human RIG-I, the oyster RIG-1 can bind to RNA, interacts with the oyster MAVS, and through its activation domains, recruits caspase and TRAF6, which subsequently activates the NF-κB signaling pathway [59]. Further studies revealed the presence of 13 RLR family proteins in the oyster *Crassostrea gigas*, 11 of which were significantly activated upon infection with herpes [61]. Other mollusks, such as *Bathymodiolus platifrons* and *Mytilus coruscus,* have 12 and 19 TLR genes, respectively (Table 3). It turned out that RLR was completely lost in arthropods in studies of 58 species [61,62].

Annotating RLRs in the genomes of 58 other protostomes, *Lophotrochozoa*, revealed a complex and unique arrangement of lophotrochosis RLR domains, which may be the result of exon-intron divergence, expression diversification and positive selection [61].

## 5. C-Type Lectin Receptors

**Figure 4:**
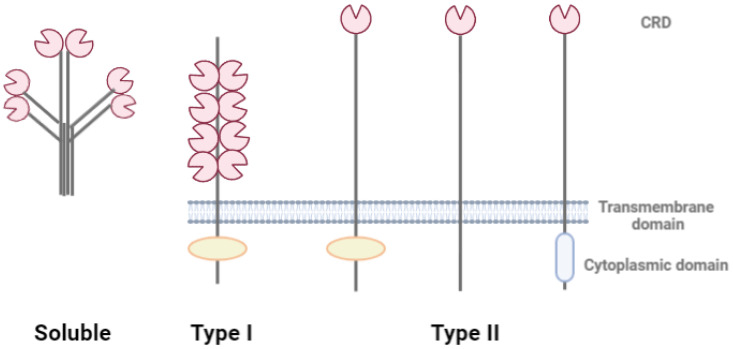
C-type lectin receptors (CLRs). Soluble CLRs have several carbohydrate recognition domains (CRDs) and have no transmembrane domain. Type I CLRs include several CRD or CRD-like domains, transmembrane and cytoplasmic domains. Type II CLRs have one CRD, transmembrane and different cytoplasmic domains.

The C-type lectin receptors (CLRs) include more than 1000 proteins of multicellular organisms, with carbohydrate recognition domains (CRDs) that bind to carbohydrates in a calcium-dependent manner [63]. Based on their structure, CLRs are unified into the following three groups: soluble, membrane-bound type I, and membrane-bound type II [64]. Soluble CLRs include mannose-binding lectin (MBL), which activates the complement system, stimulating innate immunity against yeast [65,66]. MBL recognizes mannose on microorganisms, leading to opsonization and activation of the complement lectin pathway. MBL also interacts with HIV glycoprotein (gp)120 carbohydrates and can inhibit the spread of HIV [67]. Membrane-bound lectins are divided into two large groups, depending on the number of carbohydrate recognition domains. The type I CLRs have multiple domains, while the type II CLRs have one domain (Figure 4). In this case, the cytoplasmic domain has a different structure; in particular, it can have an immunoreceptor tyrosine-based activation motif (ITAM) or immunoreceptor tyrosine-based inhibition motif (ITIM), which impart immunostimulatory or immunosuppressive functions, respectively. The ITAM motif is required for signaling to the downstream activation pathway [68]. There are 17 CLR families based on their phylogeny [69].

CLRs function as PRRs, recognizing microbial components and internalizing various glycoproteins and microbes for clearance and antigen presentation to T lymphocytes [70]. CLR-induced signaling cascades lead to activation of the nuclear factor kappa-B (NF-κB) family of transcription factors via Syk- and CARD9-dependent pathways. NF-κB activation plays a critical role in the induction of innate immune and inflammatory responses during microbial infection and tissue damage [71,72,73]. The function of CLRs is not only to recognize pathogens, but also to detect dead and transformed cells [74]. One of the CLRs expressed on macrophages recognizes the small nuclear ribonucleoprotein component, which is released from dead cells and stimulates macrophages to produce inflammatory cytokines and chemokines and to initiate phagocytosis [75].

Genomic sequencing of C-type lectin receptors has shown many invertebrate CLR proteins, with their domain architecture markedly different from vertebrates [63]. Vertebrate CLRs have evolved to specifically recognize protein, lipid, and inorganic ligands, including branch-specific snake venoms, as well as fish antifreeze and avian eggshell proteins [63]. To study the evolution of C-type lectin receptors, the approach of comparing genes of different CLR clusters is used [76,77]. The CLECT C-type lectin motif for carbohydrate recognition was found to have been emerged early in evolution. It can be found in the proteins of many model organisms, including the yeast *Saccharomyces cerevisiae*, the nematode *Caenorhabditis elegans*, the fruit fly *Drosophila melanogaster*, and the ascidian tunicate *Ciona*, as shown Table 4 [76,77,78,79].

The DECTIN-1 CLR type I cluster has significant homology with other species of organisms and is considered to have arisen in humans as a result of subsequent gene duplications with inversions in Alu sequences [76,77]. The oldest species to possess a C-type lectin-like protein is the sea squirt *Botryllus schlosseri*, a colonial chordate invertebrate. The BsCD94-1 protein is a type II transmembrane receptor, with a C-type lectin-like domain most similar to mammalian C-type lectin-like receptors. Interestingly, BsCD94-1 is expressed on a subset of *B. schlosseri* blood cells and plays a role in allorecognition [77]. It is hypothesized that the first C-type lectin-like receptor genes arose prior to the divergence of fish and tetrapods over 400 million years ago, followed by independent duplications of a common ancestral gene [80].

Currently, the genomes of 3278 species of organisms, mainly vertebrates, are known [81]. It is noted that the genomes of invertebrates, including marine ones, are less studied [81]. Chordata phylum data amounted to 1770 assemblies (54% of all assemblies), despite the fact that chordates make up only 3.9% of animal species. Conversely, invertebrates were underrepresented, with 1115 assemblies (34% of the dataset) for a group that includes 78.5% of animal species [81]. Robust study of the genome of marine invertebrates will reveal the earliest changes in innate immunity mechanisms depending on habitat and interactions with other organisms.

## 6. Cellular Factors

Implementation of the immune response in mammals is based on cellular and humoral factors of innate and adaptive immunity. Epithelial and phagocytic cells, related to innate immunity, represent the first line of defense against pathogens.

Hydra, one of the simplest known multicellular aquatic animals, was studied as an example, allowing us to trace the evolution of the innate immune system. It turned out that hydra completely lacks mobile phagocytes and Toll-like receptors (TLRs). All antimicrobial protection is carried out by the hydra epithelium, equipped with powerful antimicrobial peptides. The induction of antimicrobial peptide production in the hydra epithelium is mediated by the interaction of the proteins that contain leucine-rich repeats (LRRs) with the proteins that contain a TIR domain that lacks LRRs [82]. Based on this research, Bosch and colleagues concluded that the epithelium represents an ancient host defense system.

Phagocytosis is believed to have originated about 1800 million years ago in ancient eukaryotic organisms, while the ancestors of the simplest organisms—bacteria and archaea—that arose about 4000 million years ago, did not have phagocytic ability [83,84,85].

Hemocytes are the main motile cells of invertebrates responsible for phagocytosis and production of soluble antimicrobial and cytotoxic factors [86]. In cellular immunity of ascidia, a chordate marine invertebrate belonging to the *Tunicata* branch of the Chordate phylum, hemocytes can circulate in the hemolymph and pharynx and, upon PAMP invasion, can differentiate to produce inflammatory factors [87].

Considering the function of innate cellular immunity as not only being the response to PAMP, but also providing an ability to distinguish cells of their own body, invertebrates are a convenient model for studying the mechanisms of transplant rejection in representatives of the same species.

Sponges (the Porifera phylum) are a convenient model object for revealing the rejection mechanism. In the case of genetically homogeneous individuals growing side by side, this rejection is not observed, and their organisms can merge together, grow and multiply [88]. Using the sponge *Callyspongia diffusa* colonies, in which a graft rejection upon repeated contacts with incompatible tissues occur, an effector mechanism was found, which involved the faster release of cytotoxic proteins that destroyed foreign tissues [88,89].

The colonial tunicate ascidian *Botrillus schlosseri*, belonging to the earliest branch of the Chordata phylum, and the *Tunicata* subphylumcan fuse with other colonies with the vascular reorganization and the formation of new blood vessels. Individuals that are genetically different with regard to one allele may have an inflammatory reaction, causing rejection. The rejection reaction begins with the migration of a specific type of hemocytes, the morula cells, to the tips of the interacting colonies, where they release the contents of their vacuoles and initiate an inflammatory response that includes the formation of melanin scars, the so-called “rejection points” [90]. Both outcomes are controlled by a single fusibility/histocompatibility (Fu/HC) locus, with multiple codominantly expressed alleles.

It is known that most transplant rejections occur due to the activation of the adaptive immune response; however, the pro-inflammatory response of the innate immune system is required for the activation of adaptive immunity. The colonial tunicates *B. schlosseri* are invertebrates and are a part of the closest group to vertebrates that lack T- and B-cell-based adaptive immunity [91]. It has unique characteristics that make it a valuable model system for studying the mechanisms of innate immunity in relation to the phenomenon of natural allogeneic transplantation, which results in either fusion or rejection. When two colonies of *B. schlosseri* come into contact, they recognize each other on a friend-foe principle [92]. If they have at least one common allele of the polymorphic histocompatibility gene, the Botryllus histocompatibility factor (BHF), they merge their vessels, forming a natural parabiont [93]. When creating a common vascular system, cells can freely flow from one chimera partner to another, resembling mammalian chimerism at the somatic level. If the colonies are genetically incompatible, they undergo an immune rejection reaction, in which inflammatory and cytotoxic cells participate, creating zones of necrosis at points of contacts [93]. The cytotoxic morula (MK) cells form the basis of the rejection reaction and cytotoxicity, resembling human natural killer (NK) cells. Without inhibitory recognition of compatible BHFs, morula cells kill target cells, resulting in necrotic lesions [94]. This allorecognition is attributed to the invertebrate analog of transplantation immunity [95].

In higher vertebrates, T cells play a major role in chronic rejection, graft-versus-host disease, and pregnancy disorders [96]. Elucidation of the immune-related mechanisms of activation of these effector cells under allogeneic conditions will give us a better understanding of the way in which they bypass cytolytic activation and positively modulate the process of chronic rejection. NK cells and T cells in humans share the characteristic of allogeneic self/outsider identification and are activated either by identification of the outsider or by the absence of a “self”. In *B. schlosseri*, allogeneic rejection occurs in the same way as in vertebrates, despite the fact that its system is more based on innate immunity. The Botryllus histocompatibility factor (BHF) of *B. schlosseri* shares some common features with human MHC [90] and its recognition as its “self” results in a major inhibitory mechanism of cytotoxicity in allorecognition. The inhibitory effect of BHF on cytotoxicity, combined with observational evidence of colony fusion that shares at least one BHF allele, suggests that the mechanism of cellular toxicity during allorecognition in this tunicate is related to the “missing self” and can be compared to the NK recognition in higher vertebrates [97,98]. Allorecognition, as mentioned above, also involves human CLR orthologues, BsCD94 receptors [80]. This evidence also supports the concept that urochordate blood cells may belong to an ancestral cell population that represents the evolutionary origin of NK cells [99]. Taken together, these results demonstrate similarities in innate immune responses between *B. schlosseri* and humans at the cellular and molecular levels.

Thus, the study of marine invertebrates makes it possible to understand the mechanisms of innate immunity, as well as to trace the origin of the processes underlying adaptive immunity. Among further prospects in this direction, it is of interest to study the mechanisms of memory appearance in tunicates, when information about priming by a foreign agent is retained and rejection during a secondary contact occurs faster. Modern interdisciplinary approaches using genomics, transcriptomics, proteomics, metabolomics, systems biology, and bioinformatics provide us with hope for solving this problem [100,101,102,103].

## 7. Epithelial Proteins and Antimicrobial Peptides

Epithelial tissues—skin and mucous membranes—provide mechanical protection against pathogens. At the same time, the composition of mucosal components affects colonization resistance and provides an environment favorable for maintaining commensal microflora, which protects the body from infections, allergy and maintains homeostasis [104,105]. It is believed that mucosal surfaces first appeared about 560 million years ago in aquatic inhabitants—representatives of the type (phylum) Cnidaria [106]. This is why hydra is a convenient model object for studying the mechanisms of innate immunity, as it is one of the most ancient marine animals that developed mucous membranes [106,107,108].

Mammalian mucus contains mucin-like proteins, soluble IgA, lysozyme, and antimicrobial peptides (AMPs). Mucins are large glycosylated proteins that cover the cells of the mucosal epithelium. A study of mucin evolution using profiling searches in the NCBI protein sequence database revealed that most vertebrates have 5–6 gelling mucin genes and their genomic arrangement is conserved [109]. An exception is the frog *Xenopus tropicalis,* with a repertoire of 26 mucins of this type. RNA sequencing revealed that these proteins are widely distributed in invertebrates. Their presence in *Cnidaria, Porifera*, and *Ctenophora* (comb jellies) indicates that these proteins were present early in metazoan evolution. A conserved N-terminal FCGBP domain has been identified in various organisms, including a number of bacterial proteins [109,110]. This study demonstrates the very early origin of mucin-like proteins.

Antimicrobial peptides (AMPs) are important elements of the mucosal epithelium of invertebrates, providing immune protection [111,112]. The mechanism of implementation of the biological activity of AMPs is based on the following several strategies: (1) destruction of the bacterial membrane; (2) perforation of the bacterial membrane; (3) penetration into the bacterium and interaction with intracellular organelles [113]. AMPs are constitutively and inducibly expressed and modulate immune responses against pathogens [114]. In this context, AMPs represent the main humoral defense against infections. Marine invertebrates are constantly exposed to a huge microbial load from the aquatic environment. Over the past two decades, a great number of AMPs have been isolated from marine invertebrates, including cnidarians, molluscs, annelids, arthropods, and tunicates [114,115,116].

Cnidarians assemble the group of aquatic organisms that includes hydroids, coral polyps, box jellyfish, and scyphoids. Aurelin, exhibiting antimicrobial properties against Gram-positive and Gram-negative bacteria, and hydrolysin belong to this group [117,118].

Mollusks are the type of protostomes, including clams, mussels, squids, octopuses, polyplacophores, and gastropods. Most of their AMPs are cationic cysteine-rich peptides. According to the primary structure and types of disulfide bond, mollusc AMPs are distinguished as defensins, mytilin, myticin, and mytimycin [119,120,121]. The characterized peptides have varying levels of antimicrobial activity, some of them (myticin C) also have antiviral and immunomodulatory activity and control the development of the larval stage [122,123]. Mytimycin has antifungal properties; inhibits the growth of *Neurospora crassa* and *Fusarium culmorum* [124,125].

Annelids include worms, leeches and misostomids. AMPs isolated from this type include arenicins, perinerin, and hedistin, nicomycins, capitellacin, abarenicin. Arenicins have a wide spectrum of antimicrobial activity against fungi and bacteria [126,127,128,129,130]. At low concentrations, arenicins activate the compliment system [129]. It turned out that the dimerization of arenicin is a key moment for the cytotoxic properties of arenicin [131]. Perinerin has activity against Gram-positive and Gram-negative bacteria and fungi [132]. Hedistin has a wide spectrum of antimicrobial activity, including methicillin-resistant strains of *Staphylococcus aureus* and *Vibrio alginolyticus* [133]. Nicomycin and capitellacin, isolated from the arctic polychaeta *Nicomache minor* and *Capitella teleta,* have not only antibacterial activity, but also cytotoxicity against tumor cells [134,135]. Capitellacin destroyed biofilms and prevented the formation of new *E. coli* biofilms [136]. Abarenicin has strong antibacterial potential against a wide range of Gram-negative bacteria, including drug-resistant strains [137].

Arthropod AMPs are represented by penaeidins isolated from crustaceans (shrimps), as well as by polyphemusins and tachyplesins from horseshoe crabs [138,139,140,141]. Penaeidins showed pronounced activity against some Gram-positive and Gram-negative bacteria, as well as against filamentous fungi, and did not affect *Candida albicans* or *Saccharomyces cerevisiae* [142,143]. Tachyplesin I has a broad spectrum of an antimicrobial activity against Gram-negative and Gram-positive bacteria, fungi, and viruses [144]. The cytotoxic activity of tachyplesin I towards various tumor cells was also found [145].

Among the AMPs of tunicates, the majority were isolated from ascidian hemocytes. These are stielins, clavaspirin, clavanins, halocyamines, plicatamide, dicintaurin and halocidin [146,147,148,149,150,151]. It turned out that clavanins and plicatamide are active against methicillin-resistant strains of *Staphylococcus aureus* and are promising candidates for the development of drugs for the treatment of sepsis and wound infections [151,152].

All of the listed AMPs are constitutively expressed, regardless of the activation stimulus. In response to a microbial infection, invertebrates can produce additional antimicrobial proteins and peptides that activate defense. In the sequenced genome of the sea urchin *Strongylocentrotus purpuratus,* 17 genes were found that were activated in response to immune stress, encoding SpTrf proteins that bind to bacteria and yeast and enhance phagocytosis [153]. In the ascidian *Ciona robusta*, the inflammatory stimulus activates the expression of not only AMP genes, but also galectins, C-type lectins, collectins, interlectins, complement factor orthologues, TNFα, and IL-17 [87].

## 8. Conclusions

Preservation of homeostasis of ancient marine multicellular organisms is supported by physical barriers, epithelium, humoral and cellular factors of innate immunity. With the complexity of the structure of a multicellular organism, the variety of ways to protect against pathogens increases.

Convergent and divergent processes in the evolution of defense mechanisms against pathogens include not only changes in pathogen pattern recognition receptors specific to different types of pathogens, but also in the identification of metabolic disorders that can be caused by both pathogen invasion and tissue destruction. Evidently, the determination of metabolite-mediated pathogen invasion can potentially help to protect the body from infections not detected by TLRs or NLRs.

Investigation of the most ancient marine chordates—tunicates—gave an example of another function of the innate immunity of invertebrates. It was established that allograft rejection or tolerance to it and the formation of chimeras, depending on the absence or presence of a common allele of the BHF histocompatibility gene, took place. In humans, similar functions are performed by cells of adaptive immunity.

Protective proteins and antimicrobial peptides are present in all organisms. They are the most ancient molecular factors of innate immunity that maintain homeostasis. They can serve as the basis for the development of new drugs for the treatment and prevention of infectious diseases. A detailed study of the mechanisms of the functioning of the innate immunity system of the most ancient marine multicellular organisms provides valuable data for scientific analysis and future practical application.

## Figures and Tables

**Table 1:** The number of TLRs in different organisms.

*Hydra magnipapillata*	*Strongylocentrotus purpuratu* (Sea Urchin)	*Gadiformes morhua* (Fish)	*Homo sapiens*
0	222	42	10

**Table 2:** The number of NLRs and NLR-like proteins in different organisms.

*Hydra magnipapillata*	*Strongylocentrotus purpuratu* (Sea Urchin)	*Amphimedon queenslandica* (Sponge)	*Homo sapiens*
290 ^1^	203	135	≈20

^1^ NLR-like.

**Table 3:** The number of RLRs in different organisms.

*Amphimedon queenslandica* (Sponge Porifera)	*Crassostrea gigas* (Pacific Oyster)	*Mytilus coruscus* (Mollusca)	*Homo sapiens*
2	13	19	3

**Table 4:** The number of CLRs in different organisms ^1^.

*Botryllus schlosseri* (Ascidian Tunicate)	*Ciona* (Ascidian Tunicate)	*Homo sapiens*
1	1	≈1000

^1^ On the base of C-type lectin motif.

## Data Availability

Not applicable.
